# Methadone initiation in a bridge clinic for opioid withdrawal and opioid treatment program linkage: a case report applying the 72-hour rule

**DOI:** 10.1186/s13722-021-00279-x

**Published:** 2021-12-28

**Authors:** Jordana Laks, Jessica Kehoe, Natalija M. Farrell, Miriam Komaromy, Jonathan Kolodziej, Alexander Y. Walley, Jessica L. Taylor

**Affiliations:** 1 Department of Medicine, Section of General Internal Medicine, Boston University School of Medicine and Boston Medical Center, Boston, MA USA; 2 Grayken Center for Addiction, Boston Medical Center, Boston, MA USA; 3 Department of Pharmacy, Boston Medical Center, Boston, MA USA; 4 Department of Emergency Medicine, Boston University School of Medicine, Boston, MA USA; 5 Addiction Treatment Center of New England, Brighton, MA USA

**Keywords:** Case report, Opioid use disorder, Opioid withdrawal, Methadone, Low-barrier bridge clinic, Opioid treatment program

## Abstract

**Background:**

In the United States, methadone for opioid use disorder (OUD) is limited to highly regulated opioid treatment programs (OTPs), rendering it inaccessible to many patients. The “72-hour rule” allows non-OTP providers to administer methadone for emergency opioid withdrawal management while arranging ongoing care. Low-barrier substance use disorder (SUD) bridge clinics provide rapid access to buprenorphine but offer an opportunity to treat acute opioid withdrawal while facilitating OTP linkage. We describe the case of a patient with OUD who received methadone for opioid withdrawal in a bridge clinic and linked to an OTP within 72 h.

**Case presentation:**

A 54-year-old woman with severe OUD was seen in a SUD bridge clinic requesting OTP linkage and assessed with a clinical opiate withdrawal scale (COWS) score of 12. She reported daily nasal use of 1 g heroin/fentanyl. Prior OUD treatment included buprenorphine-naloxone, which was only partially effective. Her acute opioid withdrawal was treated with a single observed oral dose of methadone 20 mg. She returned the following day with persistent opioid withdrawal (COWS score 11) and was treated with methadone 40 mg. On day 3, the patient was successfully admitted to a local OTP, where she remained engaged 3 months later.

**Conclusions:**

While patients continue to face substantial access barriers, bridge clinics can play an important role in treating opioid withdrawal, building partnerships with OTPs to initiate methadone on demand, and preventing life-threatening delays to methadone treatment. Federal policy reform is urgently needed to make methadone more accessible to people with OUD.

## Background

Fentanyl overdose deaths have surged during the coronavirus pandemic, likely driven by isolation, disrupted life routines, and decreased access to opioid use disorder (OUD) treatment and harm reduction services. Data from the Centers for Disease Control and Prevention show 2020 opioid overdose deaths rates in most U.S. states to be the highest in history [1], including in Massachusetts [2].

Methadone, a first-line medication for OUD, reduces all-cause and overdose mortality [3], increases treatment engagement, and prevents harm related to injection drug use, including HIV and hepatitis C [4, 5]. Patients face multiple barriers to accessing methadone in the U.S. Since methadone first became widely available for OUD in the 1970s, methadone for OUD is only available to outpatients through opioid treatment programs (OTPs). The Drug Enforcement Agency and the Substance Abuse and Mental Health Services Administration license and certify OTPs, respectively, and set detailed requirements for clinic characteristics, patient eligibility, dosing standards, observed dosing, and counseling [6].

The number and capacity of OTPs nationally is insufficient [7]. Restrictive zoning regulations, insufficient insurance coverage, and understaffing contribute to inadequate capacity, which can lead to long waits for intake appointments. Rural areas are particularly underserved, and patients may need to wait more than a year and commute long distances to access an OTP [8]. Delays in treatment increase the risk of continued drug use, infectious complications, and overdose death while decreasing the likelihood of eventual treatment initiation [9]. Black individuals, persons experiencing homelessness, and those with criminal justice involvement experience even higher barriers to methadone for OUD [10, 11] contributing to racial, ethnic, and socioeconomic treatment inequities.

The “72-hour rule” (Title 21, Code of Federal Regulations, Part 1306.07(b)) provides an important exception to the requirement that methadone for OUD only be provided outpatient in licensed and certified OTPs. This exception allows physicians who are not registered as an OTP to administer “narcotic drugs to a person for the purpose of relieving acute withdrawal symptoms” for up to three days while arranging for ongoing treatment [12]. The medication must be administered directly to the patient and cannot be prescribed, and no more than one day’s medication may be administered at a time. To date, the 72-hour rule has been used primarily in emergency departments (EDs). Note that for inpatients admitted to the hospital for medical or surgical problems, there are no limits to administering methadone for withdrawal or OUD [12, 13].

Substance use disorder (SUD) bridge clinics [14–16], many of which are hospital-based, provide low-threshold, on-demand access to medications for addiction treatment and may have the clinical infrastructure needed to administer methadone for emergency opioid withdrawal management. Bridge clinics also have strong connections to community partners and expertise in linking patients to long-term OUD care. Thus far, bridge clinics have focused on low-barrier buprenorphine access. To our knowledge, there are no published examples of bridge clinic administration of methadone for emergency withdrawal management with linkage to long-term methadone treatment.

Application of the 72-hour rule in the bridge clinic setting promises benefits for patients. Rapid, low-barrier opioid withdrawal management can reduce ongoing opioid use and overdose risk, decrease avoidable ED presentations for opioid withdrawal, facilitate timely OTP referrals, and allow time to address barriers to OTP linkage. Here, we describe the first case of methadone administration for emergency opioid withdrawal treatment and direct OTP linkage in Faster Paths, a low-barrier outpatient bridge clinic at a safety net hospital in Boston, MA. The patient provided informed consent to publish this case report.

Faster Paths opened in 2016 and serves approximately 650 patients/year, offering rapid access to medications for addiction treatment; HIV, hepatitis C, and sexually transmitted infection prevention and treatment; harm reduction services; and linkage to long-term MOUD treatment and community resources [16]. At the time of this case, medication services included buprenorphine and naltrexone for OUD; naloxone for opioid overdose reversal; naltrexone, acamprosate, and disulfiram for alcohol use disorder; and outpatient medical management of opioid, alcohol, and benzodiazepine withdrawal. The clinic is staffed by internal medicine physicians, many of whom are board certified in Addiction Medicine, nurse practitioners, a nurse care manager, and rotating fellows and residents. Referral sources include the ED, local addiction treatment and harm reduction programs, word of mouth, and the inpatient Addiction Consult Service.

## Case presentation

A 54-year-old woman with a past medical history of severe OUD, major depressive disorder, and cervical radiculopathy was referred to the hospital’s SUD bridge clinic from the ED for help stopping heroin/fentanyl use. She reported a 16-year history of OUD with a previous trial of buprenorphine-naloxone that was only partially effective for cravings, prior episodes of methadone for inpatient opioid withdrawal management only, and extended periods of abstinence including eight years without any medication for OUD (MOUD), which preceded her 8-month relapse in the setting of significant stressors. Current substance use included one gram of nasal heroin/fentanyl daily with last use 15 hour prior to presentation and occasional smoked cocaine and cannabis. She reported one prior opioid overdose. Family history included severe OUD in her son and her sister, who died of a fentanyl overdose.

She was highly motivated to re-engage in treatment. Because of incomplete craving control on buprenorphine in the past, she requested methadone treatment for the first time. She reported opioid withdrawal symptoms including body aches, nausea, and malaise. On exam, her vital signs were within normal limits. She was restless during the visit with pupils 5 mm and observed rhinorrhea. A Clinical Opiate Withdrawal Scale (COWS) score was not formally assessed but estimated to be ≥ 12 based on documented symptoms and exam findings. Laboratory results, including a comprehensive metabolic panel and HIV and viral hepatitis serologies, were unremarkable.

The bridge clinic team contacted a local OTP and scheduled an intake appointment for two days later. Based on the patient’s history and presentation of acute opioid withdrawal, she was administered a single observed oral dose of methadone 20 mg in the clinic from the hospital pharmacy.

The patient returned to clinic the next morning as instructed for ongoing emergency withdrawal management. In the interim, she reported abstinence from all illicit drugs and alcohol and persistent opioid withdrawal (COWS score approximately 11). She was administered an increased observed dose of methadone 40 mg. The bridge clinic team also connected her to a recovery coach and arranged transportation for her OTP intake appointment. On day three, the patient successfully completed intake and admission procedures to the OTP, where she received a 40 mg dose of methadone. Three months after her first bridge clinic appointment, she continued to maintain excellent adherence to her daily methadone dose of 80 mg and reported satisfaction with this treatment.

## Discussion and conclusions

### Bridge Clinic administration of methadone is feasible

The National Academy of Sciences, Engineering and Medicine’s consensus report, Medications for Opioid Use Disorders Saves Lives, concludes that all FDA-approved MOUD should be available in all treatment venues [17]. Yet there are few outpatient venues where methadone, buprenorphine, and naltrexone are available. Low-barrier SUD bridge clinics have made major strides in reducing barriers to buprenorphine but cannot offer methadone for OUD unless they are licensed as OTPs. Here, we describe a novel use of bridge clinic infrastructure—administration of methadone for acute opioid withdrawal management under the provisions of the 72-hour rule—alongside rapid OTP linkage, resulting in a successful and sustained transition to methadone treatment for OUD.

Treating acute opioid withdrawal for up to 72 hour in a bridge clinic with rapid OTP linkage represents an innovative pathway that can facilitate treatment entry for patients at high risk of overdose. Through this pathway, the bridge clinic becomes a rare setting where patients can be offered the full range of MOUD options, along with a comprehensive medical evaluation and assistance with challenges such as transportation that could interfere with OTP attendance.

### Logistical and infrastructure considerations

Providing emergency withdrawal management under the 72-hour rule hinges on the ability to arrange ongoing care. In this pilot case, bridge clinic providers were able to link the patient to a local OTP that offers direct admissions in two days. Two days is still too long to wait for life-saving treatment; however, many OTPs report waits of several weeks or longer for new intakes [9]. Despite having five OTPs located in Boston, the number of methadone treatment slots per capita is lower than many Massachusetts communities, and patients typically wait two weeks or longer to initiate methadone when presenting from the community.

Given their strong community referral networks, bridge clinics are well-positioned to develop rapid referral agreements with local OTPs, who may accrue programmatic benefits from accepting patients deemed clinically appropriate for methadone who have already received up to 72 hour of withdrawal treatment. The success of this case and subsequent increase in patient demand prompted our clinic to sign affiliation agreements with five total OTPs, thus offering flexibility in OTP location.

The ability to administer methadone in a bridge clinic depends on significant programmatic and institutional resources. Our clinic collaborated with the pharmacy department to add methadone to the automated medication dispensing cabinet, which fulfills requirements for controlled substance storage, continuous inventory, and waste. Providers place methadone orders in the electronic medical record to allow for administration documentation. Administrating methadone may not be feasible for bridge clinics that lack medication storage and administration infrastructure; however, this represents a promising opportunity for hospital-based programs.

This initial case that utilized the 72-hour rule in March 2021 became a model for a new clinical pathway offering opioid withdrawal management with methadone and OTP linkage that treated 157 unique patients in its first five months. The immediate, high demand and increase in clinical volume required rapid operational adaptations, including expanding clinic hours from six to seven days/week and increasing the provider staffing pool. We plan to describe the larger patient cohort including OTP linkage and retention outcomes as the next step in evaluating and disseminating this clinical innovation.

### Fundamental policy reforms needed

This report describes an important bridge clinic innovation that can lower barriers to methadone treatment for OUD, particularly for marginalized groups. However, the significant demand for opioid withdrawal treatment and OTP linkage only underscores the unmet need for methadone treatment and the urgency of systems change. Regulatory reform is urgently needed to reduce barriers to methadone, and the Biden-Harris administration has committed to modernizing methadone policies in 2021–2022 [18]. We urge federal and state policymakers to follow the lead of countries like Australia, Canada, and the United Kingdom that have integrated methadone treatment for OUD into primary care and adopted pharmacy-based models of observed and take-home dosing [19, 20] Until federal policies are reformed, bridge clinics can play an essential role in treating acute opioid withdrawal and building partnerships with local OTPs to prevent life-threatening delays to methadone treatment.

## Data Availability

Not applicable.

## Abbreviations

COWS: Clinical opiate withdrawal score
ED: Emergency department
HIV: Human immunodeficiency virus
MOUD: Medications for opioid use disorder
OTP: Opioid treatment program
OUD: Opioid use disorder
SUD: Substance use disorder
